# Assessment of Residential History Generation Using a Public-Record Database

**DOI:** 10.3390/ijerph120911670

**Published:** 2015-09-17

**Authors:** David C. Wheeler, Aobo Wang

**Affiliations:** Department of Biostatistics, Virginia Commonwealth University, One Capitol Square, 830 East Main Street, Richmond, VA 23298, USA; E-Mail: wanga3@vcu.edu

**Keywords:** residential history, addresses, LexisNexis, environment

## Abstract

In studies of disease with potential environmental risk factors, residential location is often used as a surrogate for unknown environmental exposures or as a basis for assigning environmental exposures. These studies most typically use the residential location at the time of diagnosis due to ease of collection. However, previous residential locations may be more useful for risk analysis because of population mobility and disease latency. When residential histories have not been collected in a study, it may be possible to generate them through public-record databases. In this study, we evaluated the ability of a public-records database from LexisNexis to provide residential histories for subjects in a geographically diverse cohort study. We calculated 11 performance metrics comparing study-collected addresses and two address retrieval services from LexisNexis. We found 77% and 90% match rates for city and state and 72% and 87% detailed address match rates with the basic and enhanced services, respectively. The enhanced LexisNexis service covered 86% of the time at residential addresses recorded in the study. The mean match rate for detailed address matches varied spatially over states. The results suggest that public record databases can be useful for reconstructing residential histories for subjects in epidemiologic studies.

## 1. Introduction

There are many uncertainties when conducting research in spatial epidemiology and environmental epidemiology. Two major uncertainties are the location and timing of etiologically relevant environmental exposures that increase disease risk for individuals in a study population. In typical spatial analyses of epidemiologic data, the focus is on the location (e.g., where in space is risk elevated) with less consideration of the timing of the exposures (e.g., when and where in space was risk elevated). This is evident through the common use in risk analysis of spatial information that is related only to the time of study enrollment [1]. The spatial information is often the residential location, which is used as a surrogate for unknown environmental exposures or is used in environmental epidemiology to assign environmental exposures for potential risk factors of interest. The inherent assumption is that time of study enrollment is the relevant time of environmental exposures, or that the study population is not residentially mobile over time so that the residential location at the time of study enrollment represents the relevant environmental exposures.

However, high levels of population mobility and disease latencies make the use of addresses at study enrollment questionable for many health outcomes. According to the 2013 Annual Social and Economic Supplement (ASEC) of the Current Population Survey (CPS) conducted by the U.S. Census Bureau, 11.7 percent of people aged one year or more living in the United States changed residences between 2012 and 2013 [2], and the five-year mover rate was 35.4 percent from 2005 to 2010 and 44.1 percent from 1990 to 1995 [3]. The estimates of the five-year mover rate are underestimates of population mobility, as the five-year mobility survey question only asks if a person lives at the same residential location as five years ago. It has also been estimated based on the 2007 American Community Survey from the U.S. Census Bureau that a person in the United States on average moves 11.7 times in his/her lifetime [4]. In addition, the median duration of residence in the U.S. in 1996 was only 4.7 years [5]. Moreover, simulation studies show that the levels of population mobility in the United States are sufficient to obscure the spatial signal related to pertinent, historic environmental exposures for diseases with long latencies [6,7]. In addition, the power to detect an area of elevated risk and the spatial sensitivity of detection models both decrease when population mobility is simulated [8].

When a disease has a long latency, or lag time between exposure to an important risk factor and diagnosis of chronic disease, the relevance of the residential location at time of study enrollment may be minimal. For certain cancers, the latency period can be substantial. For example, the latency for cancers such as lung and bladder has been estimated to be between 20 and 30 years [9,10], while the latency for mesothelioma is estimated to be between 20 and 50 years [11]. For these diseases, and others with long latencies, spatial epidemiologic studies need to consider residential locations over a long time period for study subjects, and allow for the possibility of different environmental exposures at each residential location.

Once residential histories are collected in a study, historic patterns of spatial risk can be assessed [12,13] and historic environmental exposures can be assigned in studies [14,15,16]. However, few published epidemiologic studies in the United States have collected residential histories for subjects. An option when address histories have not been collected is to consider purchasing residential histories from public record database providers. Previous work has explored this option and compared residential history data obtained through a survey in a case-control study of bladder cancer to those available from a public-record database sold by LexisNexis using five performance metrics [17]. The previous study used data for 946 individuals from a case-control study with enrollment limited to subjects living in one of 11 counties in Michigan for at least five consecutive years [17]. In addition, the previous study limited the address lookup from the LexisNexis database to only up to the three most recent addresses per subject. Our aim in this paper was to expand on previous research and evaluate the ability of a public record database from LexisNexis to replicate address histories recorded during follow-up in a geographically diverse cohort study using a large set of performance metrics and multiple public-record database products, including an address query designed to cover the entire cohort follow-up period.

**Figure 1:**
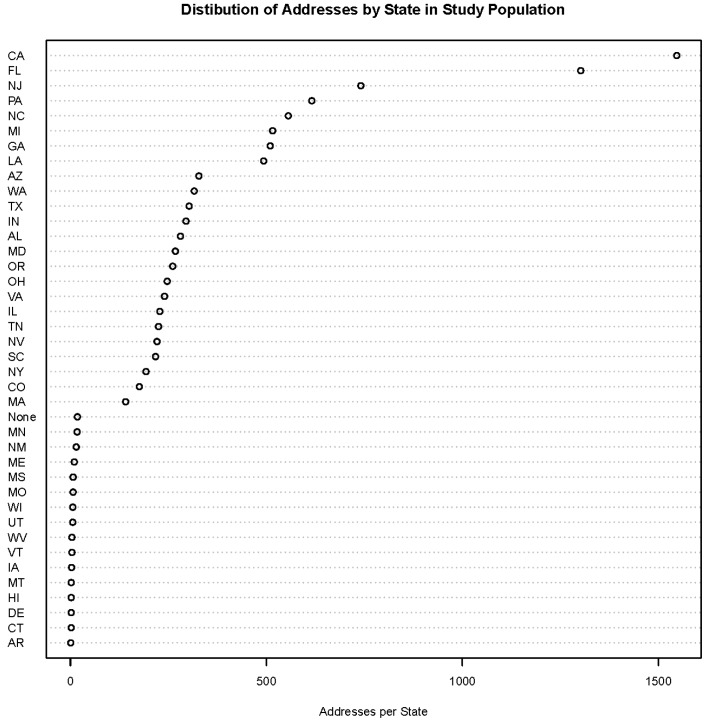
Distribution of study population addresses by state.

## 2. Methods

### 2.1. Study Population

The study population was a random sample of 1000 subjects enrolled in the National Institutes of Health-American Association of Retired Persons (NIH-AARP) Diet and Health Study, which is a large cohort study of over 560,000 AARP members aged 50–69 years and living in one of six specific states (California, Florida, Pennsylvania, New Jersey, North Carolina, and Louisiana) or two metropolitan areas (Atlanta, Georgia, and Detroit, Michigan) at the time of enrollment [18,19]. A total of 10,327 residential addresses were recorded over the course of follow-up for these subjects. The addresses started with the baseline address at the time of study enrollment, either 1995 or 1996 for subjects, and reflected address updates through 2013 as they became available from vendors who used the National Change of Address product from the United States Postal Service (USPS), which is based on the USPS change-of-address form data. The year that was recorded was the address update year and not necessarily the address change year. The distribution of all the study population addresses by state is shown in Figure 1. The top eight rows in the dot chart correspond to study enrollment states.

### 2.2. Public-Records Database

To generate residential histories for study population subjects, we placed queries with the public-records database provider LexisNexis for subject addresses. LexisNexis has compiled more than 45 billion public records from more than 10,000 diverse sources since 1991 and has one of the largest collections of online public records [20]. We used two public records services from LexisNexis. The basic service was to return up to the last three known addresses for each subject. An enhanced service was to return the known addresses back in time to at least 1995 to cover the time since enrollment in the cohort study. For both services, we provided LexisNexis with subject names and the one or two most recent addresses recorded in the NIH-AARP Diet and Health Study (some subjects had only one address recorded).

### 2.3. Address Matching

To match the study addresses and the database addresses, we developed computer programs to allow for approximate string matching in the computing environment R [21] (R Development Core Team 2008). We used approximate string matching of addresses to account for typographical errors and different abbreviations of place names. The approximate string matching function performs string matching using the generalized Levenshtein edit distance, which is the minimal number of insertions, deletions and substitutions needed to transform one string into another. We selected the Levenshtein edit distance parameter from a set of candidate values to minimize the error rate when determining the number of unique address city names for each subject in a sample of the study population. For this process, we manually through visual inspection determined the correct number of unique city names in the addresses for each subject in the sample and set this as the target. We then calculated the error rate in the estimated number of unique city names using string matching with each candidate parameter value. In this setting, an error would occur when two city names that were actually the same were estimated to be different or when two city names that were actually different were estimated to be the same. Exact string matching (parameter value of zero) had the highest error rate among the values considered for the Levenshtein edit distance. We used the error-minimizing Levenshtein edit distance in the calculation of all assessment metrics that were based on address string matching.

### 2.4. Address Matching Assessment

To assess the ability of the public-records database to recreate address histories recorded for the study population, we calculated 11 different metrics based on comparing address component strings, times spent at addresses, or geocoded addresses (Table 1). The first three metrics are based on matching specific address components and are similar to metrics used in another study [17]. Metric 1 is the proportion of the study addresses that had a match on the city and state name in LexisNexis. Metric 2 is the proportion of the study addresses that had a match on the city, state, and street name in LexisNexis. Metric 3 is the proportion of the study addresses that had a match on the city, state, street name and street number in LexisNexis, with these four elements matched separately. We used exact matching to match the address house number, while for city, state, and street name we used the approximate string matching described in Section 2.3. An important difference between our implementation of Metrics 1–3 and the previous study [17] is the direction of the comparison. Our study reports the proportion of study addresses that have a match in LexisNexis, whereas the previous study reports the proportion of LexisNexis addresses that match with the study addresses.

**Table 1:** Eleven metrics used to evaluate the agreement between addresses and times collected in the study population and those reported by LexisNexis.

Metric	Title	Description
1	City match	Study city and state match LexisNexis
2	Street match	Study city, state, and street name match LexisNexis
3	Detailed match	Study city, state, street name, and address number match LexisNexis
4	Years at address	Comparison of distribution of time at each address from each data source
5	Years at matched address	Proportion of study reported time covered by LexisNexis for matched addresses
6	Distribution of difference in time	Difference in time spent at each matched address from two data sources
7	Time covered	Mean proportion of study subject time covered by LexisNexis
8	Most recent address match	Study most recent address matches LexisNexis
9	Baseline address match	Study baseline address matches LexisNexis
10	Match by year of follow-up	Percent of study addresses that match LexisNexis by year
11	Spatial match	Proportion of study points with LexisNexis point within 100 ft

For each of the first three metrics, we calculated an overall match rate over all records and a mean match rate over subjects. Suppose there are n subjects, and each subject has$\;n_{i}$ unique addresses (i=1,…, n). Duplicated addresses for a subject in the study data were collapsed to derive the set of unique addresses for each subject. There are total N addresses in the survey, where $\;N=\overset{n}{\underset{i=1}{\sum }}n_{i}$. Let $m_{i}$ represent the match status for a unique address (1 if the *i*th study address has a match in LexisNexis, 0 otherwise). Using this notation, the overall match rate over all records and mean match rate over subjects are defined as

(1) $$\text{overall match rate}=\frac{\sum_{i=1}^{N}m_{i}}{N}$$

(2) $$\text{mean match rate}=\;\frac{\sum_{i=1}^{n}\frac{\sum_{j=1}^{n_{i}}m_{j}}{n_{i}}}{n}$$

Metrics 4–10 consider the times reported for addresses in the study and LexisNexis. The addresses from LexisNexis are reported with a first seen and last seen month and year, and the study addresses were reported in sequence with a first seen year. Metric 4 compares the distribution of time in years reported for each address in each data source. Metric 5 is the proportion of study reported time in years covered by LexisNexis for matched addresses. This proportion is reported for both the overall match rate over all matched addresses and the mean match rate over subjects. Metric 5 was based only on study addresses that had a match in LexisNexis. Metric 6 is the distribution of the differences in time in years reported at each matched address from the two data sources $\left(t_{i,LEXISNEXIS}-t_{i,study}\right)$. We describe the distributions in Metrics 4 and 6 through quartiles and a histogram. Metric 7 is the mean proportion of study subject time covered by LexisNexis. In contrast to Metric 5, Metric 7 was based on all of the study addresses to assess how well the public-records database could account for all study reported time at addresses. Metric 8 is the proportion of the most recent study address for all subjects that had a match in LexisNexis. Metric 9 is the proportion of the study baseline year (1995 or 1996) address for all subjects that had a match in LexisNexis. Metric 10 is the proportion of study addresses that had a match in LexisNexis by year of follow-up. Metrics 5 through Metric 10 used the detailed match of Metric 3 when matching addresses. For calculating Metrics 4 to 10, the LexisNexis data were limited to the time period 1995–2013 to correspond to the study follow-up period.

In addition to the previous metrics, we calculated a Metric 11 based on a spatial match of geocoded LexisNexis addresses with each study address. To do so, we geocoded the study and LexisNexis addresses independently using the same process and settings in ESRI Business Analyst 10.1 software to convert the addresses to spatial points on the U.S. street network. Regarding the quality of the geocoding, 90% of both the study addresses and LexisNexis addresses were geocoded at the address point or street address level. More than 98% of both the study addresses and LexisNexis addresses could be geocoded. For each study spatial point, we determined if there was a matching point from LexisNexis within 100 feet for the subject. Metric 11 is the proportion of study points that had a matching LexisNexis point within the distance threshold. We deemed 100 feet a reasonable search distance to consider the points to be on the same property. Larger thresholds produced similar results as the 100-foot threshold.

To determine if metrics varied over geography, we also stratified the data for calculation of some of the metrics. We selected two stratifications of interest: California *vs.* non-California, and Los Angeles *vs.* non-Los Angeles. These two locations were selected due to concerns about address matching quality in these areas. Moreover, California had the most study addresses of any state and Los Angeles County had the most study addresses of any county. In addition to the stratified analysis, we also calculated and mapped Metric 3 by each U.S. state.

## 3. Results

The mean match rate and overall match rate for Metrics 1, 2, 3 and 5 for the basic and enhanced address services are shown in Table 2. The mean match rate over subjects was higher than overall match rate over all records for each service for Metrics 1–3. For the basic service, the mean match rate over subjects of city and state names was 77.1%, while the overall match rate of city and state names was lower at 73.4%. As expected, the mean street match (72.5%) and detailed match (72.0%) metrics had lower match rates than the mean city and state match metric. For Metric 5, the mean match rate of subject reported time for detailed matched addresses covered by LexisNexis was 89.2% and the overall match rate of subject reported time for detailed matched addresses covered by LexisNexis was 91.0%. The enhanced service improved on all the match rates for Metrics 1–3. The overall match rate increased from 73.4% to 88.1% (difference = 14.7) for Metric 1, from 68.5% to 86.4% (difference = 17.9) for Metric 2, and from 67.9% to 85.9% (difference = 18.0) for Metric 3. For Metric 5, the enhanced service yielded slightly lower mean (88.4%) and overall (89.0%) match rates for subject reported time than the basic service.

**Table 2:** Mean subject match rate and overall match rate for Metric 1 of city and state match, Metric 2 of street match, Metric 3 of detailed match, and Metric 5 of years spent at matched address.

Metric	Title	Basic Service		Enhanced Service	
		Mean Match Rate	Overall Match Rate	Mean Match Rate	Overall Match Rate
1	City and state match	77.1%	73.4%	90.0%	88.1%
2	Street match	72.5%	68.5%	87.7%	86.4%
3	Detailed match	72.0%	67.9%	87.3%	85.9%
5	Years at matched address	89.2%	91.0%	88.4%	89.0%

The results for Metric 4 of the distribution of time spent at each address from each data source are shown in Table 3. The length of time spent at each address from the basic LexisNexis service was longer than that from the study. The mean time spent at each address from the basic LexisNexis was 9.2 years, while the mean time was 7.2 years for the study. The median (9 years) and third quartile (14 years) of time reported by the basic LexisNexis service were three years longer than those from the study. The enhanced LexisNexis service better matched the distribution of time recorded in the study, as evidenced by the mean time of 8.5 years and median time of 8 years, which was closer to the study median of 6 years. Both LexisNexis services had a minimum time of one year and a maximum time of 19 years, where the maximum time matches the restricted the time frame of the study (1995 to 2013).

**Table 3:** Results for Metric 4 of distribution of time spent in years at each address from each data source.

Data Source	Percentiles					Mean
	Min	25%	50%	75%	Max	
Basic LexisNexis	1	3	9	14	19	9.2
Enhanced LexisNexis	1	3	8	13	19	8.5
Study	0.3	3	6	11	18.5	7.2

**Figure 2:**
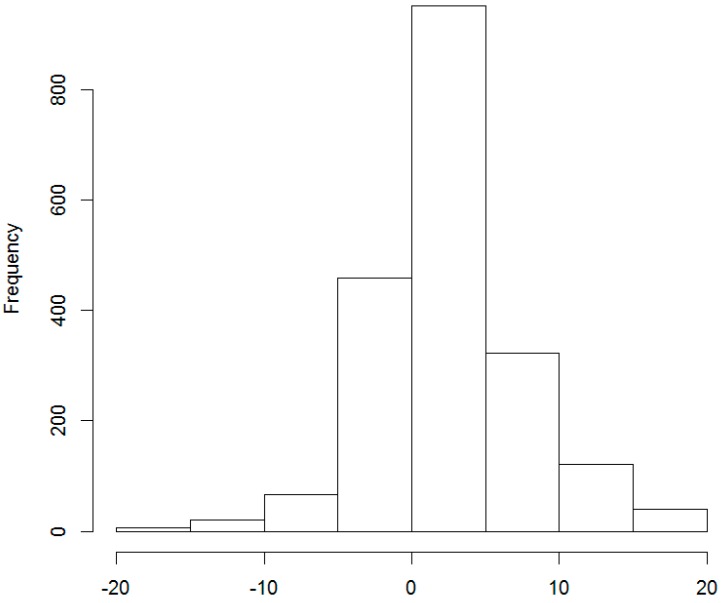
Distribution of the differences in time spent in years at each detailed matched address as reported in the enhanced LexisNexis and the study $\left(t_{i,LEXISNEXIS}-t_{i,study}\right)$.

The results for Metric 6 of the difference in time spent at each detailed matched address for LexisNexis and the study data are shown in Table 4. The distributions of differences in time spent at each detailed matched address were similar for the basic and enhanced LexisNexis services. The mean time spent at each matched detailed address from basic LexisNexis was 2.8 years longer than that from the study, while the mean difference for the enhanced LexisNexis was 2.9 years. The first quartile (0 years), median (2 years), and third quartile (5 years) of the distribution of time differences were the same for the basic and enhanced LexisNexis services. The histogram of the differences in time spent at each detailed matched address for the enhanced LexisNexis and the study data is shown in Figure 2. The mass of the distribution lies between 0 and 5 years.

**Table 4:** Results for Metric 6 of the differences in time spent in years at each detailed matched address $\left(t_{i,LEXISNEXIS}-t_{i,study}\right)$.

Data Source	Min	25%	50%	75%	Max	Mean
Basic LexisNexis	−16	0	2	5	18	2.8
Enhanced LexisNexis	−16	0	2	5	18	2.9

**Table 5:** Mean match rate for Metric 7 of time covered rate, Metric 8 of most recent address match, and Metric 9 of baseline address match for the basic and enhanced LexisNexis products.

Metric	Description	Basic LexisNexis Rate	Enhanced LexisNexis Rate
7	Time covered rate	73.8%	86.3%
8	Most recent address match	85.3%	90.5%
9	Baseline address match	53.3%	78.3%

The match rates for Metrics 7, 8, and 9 are shown in Table 5. The mean proportion of subject time covered by LexisNexis (metric 7) was 73.8% for the basic service and 86.3% for the enhanced service. The match rate for the most recent address recorded for each subject was 85.3% for the basic service and 90.5% for the enhanced service. Study baseline addresses (1995/1996) were matched by LexisNexis at 53.3% for the basic service and 78.3% for the enhanced service.

The percentage of study addresses with matches in the basic and enhanced LexisNexis by year of follow-up for Metric 10 is shown in Table 6. The match rates were above 80% for year 2004 through 2013 for the basic LexisNexis service. There was a substantial dip in the match rate from 2002 to 2001 (74.5% to 55.8%), and the match rate more gradually decreased until 1995 (40.0%). With the enhanced service, the match rate remained above 78% for all years, and the match rate was higher than the basic service match rate for every year. The annual match rate with the enhanced service was at least 20 percentage points higher than with the basic service during 1995–2001.

**Table 6:** Percent of detailed study addresses that matched basic and enhanced LexisNexis by year of follow-up (Metric 10).

Year	Count	Basic LexisNexis Match	Enhanced LexisNexis Match
1995	30	40.0%	83.3%
1996	1024	53.0%	78.3%
1997	1005	53.5%	78.5%
1998	1012	53.9%	79.3%
1999	1009	54.5%	79.4%
2000	1011	54.8%	79.8%
2001	1017	55.8%	79.9%
2002	1098	74.5%	86.2%
2003	1053	78.0%	85.9%
2004	1116	80.8%	87.4%
2005	1157	83.1%	88.9%
2006	1108	81.9%	87.6%
2007	1167	84.6%	89.8%
2008	1025	80.4%	86.2%
2009	1067	83.5%	89.1%
2010	830	84.0%	89.8%
2011	827	84.3%	90.0%
2012	897	85.7%	90.6%
2013	825	88.4%	93.3%

For Metric 11, the overall record match rate and mean subject match rate based on the spatial distance threshold of 100 feet (match based on presence of LexisNexis point within distance threshold of study point) were 68.2% and 72.0%, respectively, with the basic LexisNexis service. With the enhanced LexisNexis service, the overall record match rate and mean subject match rate were 86.6% and 88.2%, respectively.

**Figure 3:**
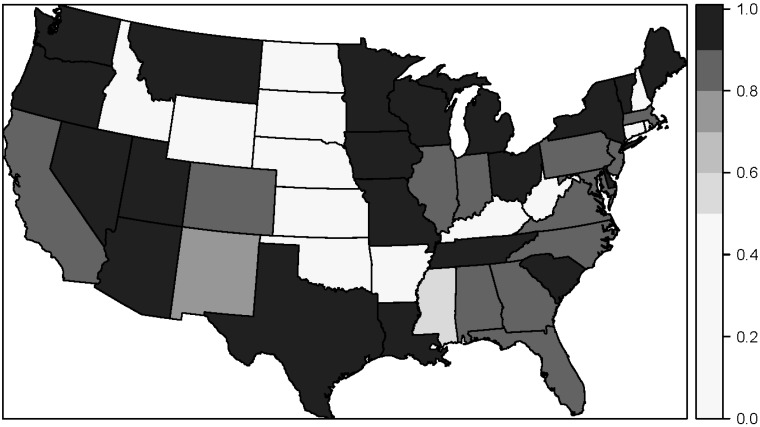
Detailed street match rate (Metric 3) by state using the enhanced LexisNexis service.

To assess the spatial dimension of the agreement between the study and LexisNexis addresses, we plotted the mean match rate by state for Metric 3 using the enhanced LexisNexis service (Figure 3). The spatial pattern of Metric 3 reveals that Midwestern states generally had higher match rates than southeastern states. For example, Ohio (91.2%) and Michigan (90.5%) had higher match rates than Alabama (84.4%) and Georgia (86.0%). Many western states, such as Washington (96.8%) and Oregon (98.4%) also had high match rates. States with a match rate of 0% had no or very few study addresses. In addition to the visual variation in match rates, the stratified analysis by California and Los Angeles showed unequal match rates among the strata. For example, with the basic service the mean match rate for Metric 3 was 58.8% for California and 74.8% outside California, and it was 62.5% for Los Angeles and 72.4% outside Los Angeles. Using the enhanced LexisNexis service the differences between strata were smaller, with a mean match rate for Metric 3 of 84.2% for California and 87.8% outside California and 85.5% for Los Angeles and 87.5% outside Los Angeles.

## 4. Discussion

In this study, we evaluated the ability of a public-records database from LexisNexis to provide residential histories for subjects in a geographically diverse cohort study. We calculated 11 performance metrics to assess the agreement between addresses collected in the study and addresses from two LexisNexis services. We found match rates of 77% and 90% for city and state together and match rates of 72% and 87% for detailed addresses with the basic and enhanced LexisNexis services, respectively. The basic and enhanced LexisNexis services were able to account for 74% and 86%, respectively, of the time at residential addresses recorded in the study. The enhanced LexisNexis product better matched the distribution of time spent at each study address than did the basic product. In addition, the enhanced product had much higher annual match rates (20 percentage points or more) of detailed addresses for years 1995–2001.

The overall better performance of the enhanced LexisNexis service compared with the basic service was expected. As the enhanced LexisNexis service was specified to provide addresses going back in time until at least 1995 and the basic service included only the three most recent addresses, we anticipated that the annual match rate for the enhanced service would be better for earlier years. Given the level of population mobility in the U.S., with a median duration of residence of 4.7 years in 1996, the three most recent addresses will not provide all the actual addresses over a 19-year period for many study subjects. The implication of this is that an enhanced service should be preferred when a project budget allows it, particularly for studies of a long duration. However, the results also suggest that the basic LexisNexis service may be adequate for studies of a short duration, as mean subject match rates for city and state and detailed addresses were respectable and the match rates by year were similar for the basic and enhanced services for more recent years (2005–2013). There were, however, relatively larger gaps between the two services for overall record match rates for Metrics 1–3. Hence, how good a substitute the basic service is for the enhanced service also depends on the type of the metric considered and if it is deemed more important to match all address records equally or maximize the average match rate per subject.

In addition to the variation observed in match rates by year, there was variation in match rates over space. The mean match rate for detailed address matches varied spatially over states, where states in the West and Midwest generally had higher match rates than states in the Southeast. Results comparing the match rates in California *vs.* out of California showed that the match rate was substantially worse in California than elsewhere in the study. The same was true for Los Angeles *vs.* outside Los Angeles. The implication of these findings is that the ability of a public records database to recreate study addresses will depend on the geographic definition of the study.

There are several strengths and limitations of this study. A strength of this study is that it includes geographically diverse addresses that cover a large portion of the United States. Enrollment into the study cohort took place in eight states, which is considerably larger than a previous study that had enrollment limited to 11 counties in Michigan [17]. In addition to the eight enrollment states, there were many other states with hundreds of addresses (Figure 1). Another strength is that our study determined how well a public-record database would recreate addresses collected in a cohort study, which more reflects how one might use the service from LexisNexis in the absence of collected residential histories. A previous study determined how well study addresses matched the LexisNexis addresses [17]. A limitation of this study is that our reported match rates may not represent those found in other studies. The cohort addresses were collected through the USPS change-of-address program. Studies based on subject recall may have lower match rates. In addition, we used approximate string matching and studies that use exact string matching may have lower match rates. While our study was geographically diverse, studies with larger sample sizes are possible with appropriate budgets.

## 5. Conclusions

Our results with this study suggest that the public record database LexisNexis can be useful for reconstructing residential histories in other studies. The usefulness of the service may depend on the beginning year of the period of interest, the duration of the period, and the geographic area of the study. More analysis should be conducted in other study populations to get a more comprehensive assessment of the ability of public record databases to recreate residential histories in epidemiologic studies.

## References

[1] Boscoe F.P., Maantay J., McLafferty S. (2011). The use of residential history in environmental health studies. Geospatial Analysis of Environmental Health.

[2] Ihrke D. (2014). Reason for Moving: 2012 to 2013. Current Population Reports.

[3] Ihrke D.K., Faber C.S. (2012). Geographical Mobility: 2005 to 2010. Current Population Reports.

[4] U.S. Bureau of the Census (2014). Calculating Migration Expectancy Using ACS Data. https://www.census.gov/hhes/migration/about/cal-mig-exp.html.

[5] Schacter J.P., Kuenzi J.J. (2002). Seasonality of Moves and the Duration and Tenure of Residence: 1996.

[6] Wheeler D.C., Calder C.A., Lawson A., Banerjee S., Haining R., Ugarte L. (2015). Socio-spatial epidemiology: Residential history analysis. Handbook of Spatial Epidemiology.

[7] Manjourides J., Pagano M. (2011). Improving the power of chronic disease surveillance by incorporating residential history. Stat. Med..

[8] Wheeler D.C., Siangphoe U., Kanaroglou P., Delmelle E., Paez A. (2015). Modeling spatial variation in disease risk in epidemiologic studies. Spatial Analysis in Health Geography.

[9] Archer V.E., Coons T., Saccomanno G., Hong D. (2004). Latency and the lung cancer epidemic among United States uranium miners. Health Phys..

[10] Miyakawa M., Tachibana M., Miyakawa A., Yoshida K., Shimada N., Murai M., Kondo T. (2001). Re-evaluation of the latent period of bladder cancer in dyestuff-plant workers in Japan. Int. J. Urol..

[11] Lanphear B.P., Buncher C.R. (1992). Latent period for malignant mesothelioma of occupational origin. J. Occup. Med..

[12] Vieira V., Webster T., Weinberg J., Aschengrau A., Ozonoff D. (2005). Spatial analysis of lung, colorectal, and breast cancer on Cape Cod: An application of generalized additive models to case-control data. Environ. Health.

[13] Wheeler D., de Roos A., Cerhan J., Morton L., Severson R., Cozen W., Ward M. (2011). Spatial-temporal analysis of non-Hodgkin lymphoma in the NCI-SEER NHL case-control study. Environ. Health.

[14] Khaw F., Pearce M., Charlton M., Pless-Mulloli T. (2004). Developing a model to estimate individual long-term exposure to air pollutants. Epidemiology.

[15] Nuckols J.R., Beane Freeman L.E., Lubin J.H., Airola M.S., Baris D., Ayotte J.D., Taylor A., Paulu C., Karagas M.R., Colt J. (2011). Estimating water supply arsenic levels in the New England Bladder Cancer Study. Environ. Health Perspect..

[16] Sørensen M., Hvidberg M., Andersen Z.J., Nordsborg R.B., Lillelund K.G., Jakobsen J., Tjønneland A., Overvad K., Raaschou-Nielsen O. (2011). Road traffic noise and stroke: A prospective cohort study. Eur. Heart J..

[17] Jacquez G.M., Slotnick M.J., Meliker J.R., AvRuskin G., Copeland G., Nriagu J. (2011). Accuracy of commercially available residential histories for epidemiologic studies. Am. J. Epidemiol..

[18] Schatzkin A., Subar A.F., Thompson F.E., Harlan L.C., Tangrea J., Hollenbeck A.R., Hurwitz P.E., Coyle L., Schussler N., Michaud D.S. (2001). Design and serendipity in establishing a large cohort with wide dietary intake distributions. Am. J. Epidemiol..

[19] Reedy J., Wirfalt E., Flood A., Mitrou P.N., Krebs-Smith S.M., Kipnis V., Midthune D., Leitzmann M., Hollenbeck A., Schatzkin A. (2010). Comparing 3 dietary pattern methods-cluster analysis, factor analysis, and index analysis-with colorectal cancer risk. Am. J. Epidemiol..

[20] LexisNexis. http://www.lexisnexis.com/en-us/products/public-records.page.

[21] R Development Core Team (2008). R: A Language and Environment for Statistical Computing.

